# Editorial Notes

**Published:** 1929

**Authors:** 


### Editorial Note.

**Hospital Rag ended.**

THE undergraduates of the University have resolved, after several discussions in the Union, to stop their annual collection for the Lord Mayor's Fund known as the "Hospital Rag."

The finances of our voluntary hospitals can ill afford to lose the contributions which have in the past been raised by this University effort. But the decision of the University Union is well-considered and fully justified. The mere title "Hospital Rag" probably offended more than it attracted, and it was calculated to bring unmerited reproach on the undergraduate collectors.

The time and energy expended by the officers of the Union in arranging the "Rag" were greater than any responsible authority should have dreamt of asking. If the Lord Mayors of this city had had any inkling of the dislocation of work and athletics caused by these preparations they would never have consented to be responsible for such a disturbance of routine.

An increasingly poor response to the collectors' appeals showed that public opinion in Bristol is out of sympathy with attempts at mass-coercion, which may sometimes succeed in less sophisticated communities. Moreover, the President of the Union was brought to death's door by illness contracted as the direct result of overwork and exposure before and during the "Rag." Mr. Cook is heartily to be congratulated on his recovery and the Union on the decision it has taken.

It remains for the next Lord Mayor to discountenance any revival of the "Rag" in other guise or under other name. There is no possible justification for imposing on the undergraduates' good nature except, perhaps, the obvious advantages that with their good nature is combined impeccable honesty, and that they did not even deduct commission from their collections.

### Obituary.

